# Sociodemographics, Comorbidities, Healthcare Utilization and Work Productivity in Japanese Patients with Adult ADHD

**DOI:** 10.1371/journal.pone.0132233

**Published:** 2015-07-06

**Authors:** Eiji Kirino, Hideyuki Imagawa, Taro Goto, William Montgomery

**Affiliations:** 1 Department of Psychiatry, Juntendo University Shizuoka Hospital, Izunokunishi City, Shizuoka, Japan; 2 Eli Lilly Japan K.K., Chuo-ku, Kobe City, Hyogo, Japan; 3 Eli Lilly Australia Pty Ltd, Sydney, Australia; Old Dominion University, UNITED STATES

## Abstract

**Objectives:**

This study compared the sociodemographic characteristics, comorbidities, healthcare resource utilization, and work productivity among Japanese adults who reported being diagnosed with attention-deficit/hyperactivity disorder (ADHD) to those of a non-ADHD control population.

**Methods:**

Data for this study were captured from an online survey of adults in Japan conducted by Kantar Health using consumer panels. A total of 84 survey participants reported they had received a diagnosis of ADHD from a physician. Survey responses pertaining to functional status and resource utilization from this ADHD group were compared to those from a non-ADHD control group of 100 participants. Comparisons between the ADHD and non-ADHD groups were made using chi-square tests for categorical variables and t-tests for continuous variables.

**Results:**

Participants in the ADHD group were on average slightly younger with a higher proportion of males. ADHD respondents reported significantly more comorbid depression, sleep difficulties, headaches, and anxiety than non-ADHD controls. Over the previous 6 months, the ADHD group made more visits to healthcare providers and the emergency room, and had more hospitalizations than non-ADHD controls. The ADHD group also rated their overall health status lower than the non-ADHD control group. Respondents with ADHD reported a significantly higher degree of health-related work impairment compared to non-ADHD, with greater absenteeism and decreased work productivity. The ADHD group indicated their symptoms negatively impacted relationships, self-esteem, and regular daily activities.

**Conclusions:**

Japanese adults with ADHD face a substantial burden of illness, including lower overall health status, increased number of comorbidities, greater healthcare utilization, and significant health-related occupational impairment compared to those without ADHD. Additional research is needed to develop a better understanding of both the consequences and treatment approaches for Japanese adults with ADHD.

## Introduction

Attention-deficit/hyperactivity disorder (ADHD) is characterized as a pattern of inattention and/or impulsivity-hyperactivity, starting in early childhood, that is more persistent, frequent, and severe than typically observed at a similar stage of development in others [1]. This disorder can manifest as predominantly inattentive or hyperactive-impulsive, or a combination of both.

### Prevalence

ADHD is a prevalent disorder that affects an estimated 4% to 7% of children worldwide [2]. While ADHD was historically thought to be predominantly a childhood disorder that remitted through adolescence, more recent data suggest that in many cases ADHD, or ADHD symptoms, tend to persist into adulthood [2–4]. In particular, the symptoms associated with inattention appear to be the most common among adults [2,5,6]. A multi-country study that included Belgium, Colombia, France, Germany, Italy, Lebanon, Mexico, the Netherlands, Spain, and the United States, found the prevalence of ADHD in adults age 18 to 44 ranged from 1.2% to 7.3% [7]. The mean estimated prevalence of adult ADHD across all 10 countries was 3.4% [7]. In Japan, the prevalence of ADHD among adults has been estimated at 1.7% [8].

### Impact of ADHD

#### Social and occupational impact

Outside of Japan, reports indicate that adults with ADHD frequently present with impairment at school, in social environments, and in the workplace [3,4,9,10]. Statements made during interviews and focus groups of adults with ADHD conducted across 7 countries in North America and Europe, indicated that participants believed general difficulties in school, rather than discrete events, had negatively impacted their life [4]. The general sentiment of participants was that appropriate diagnosis and treatment would have resulted in greater success in school, and subsequently, their careers [4]. In this same study, difficulties were reported across many different relationships, including those with parents, partners, and friends. Participants attributed these difficulties to symptoms of ADHD, such as irritability, inattention, impulsive talking, and forgetfulness [4].

In the workplace, adult ADHD has been shown to impair and reduce productivity, and also to lead to behavioral issues as a result of irritability and low frustration tolerance [10]. In addition, adults with ADHD are at an increased risk of workplace injuries [10].

An increased risk of serious vehicular accidents in adults with ADHD has also been reported [11]. In one study, over 17,000 adults with a diagnosis of ADHD were observed over a 3-year period for serious accidents, as documented in the Swedish national registers [11]. The results suggest that adults with ADHD are approximately 1.5 times more likely to be involved in a serious vehicular accident, when compared with individuals without ADHD.

#### Burden of ADHD

In addition to the related impairments in social and occupational functioning, ADHD in adults is also associated with an increased burden on the healthcare system. A study conducted in the United States comprehensively reviewed the literature for studies reporting incremental costs related to ADHD over a 20-year period [12]. This study reported that annual national incremental cost estimates for ADHD in adults ranged from $16 billion to $50 billion, indicating high rates of healthcare resource utilization in this population [12]. Furthermore, a population-based Australian study investigated a group of middle-aged adults who self-reported symptoms of inattention and hyperactivity [6]. This study found a substantial burden associated with ADHD symptoms in this population. In particular, significant associations were observed between the presence of ADHD symptoms, and depression and anxiety, as well an overall decrease in subjective health and wellbeing [6].

In Japan, adult ADHD had been less recognized in the field of psychiatry, but this has recently started to change with the approval of medication used to treat this disorder [13]. While international research exists, the impact and consequences of ADHD among the adult population in Japan have not been well studied. One study reported that, when compared to a control group, Japanese individuals diagnosed with ADHD were more likely to report experiencing stress and visiting a healthcare provider more frequently [8]. This present study compared the sociodemographic characteristics, comorbidities, healthcare resource utilization, and work productivity among Japanese adults who reported a diagnosis of ADHD to those of a non-ADHD control population.

## Materials and Methods

### Study Design

Data for this study were captured from the National Health and Wellness Survey (NHWS), administered in 2012 by Kantar Health to the adult population of Japan. The NHWS is a self-reported, Internet-based survey that assessed information on approximately 160 different conditions. These data were drawn from a larger study that also collected survey data across Europe, Australia, and the United States [14]. All respondents provided informed consent, and the protocol and questionnaire for the National Health and Wellness Survey were reviewed and approved by Essex IRB (Lebanon, NJ). The de-identified data provided to the authors was in aggregate form. The NHWS data used in this study are made available upon request for noncommercial research and validation purposes.

Two cohorts were defined for comparison; an ADHD group and a non-ADHD control group. Respondents from Japan were included in the ADHD group if they indicated they had received a diagnosis of ADHD from a physician (n = 84). The control group was comprised of 100 participants from Japan, drawn randomly from the entire sample of respondents, who indicated they did not have experience with ADHD and had not received a diagnosis of ADHD (n = 100).

### Measures

Measures were included to assess domains pertaining to comorbidities, general health, functioning, work productivity, general activity and use of healthcare services for both cohorts. Comorbidities were assessed based on self-report. Participants were asked to select which health conditions they had experienced in the past 12 months, from a list provided. The general health of participants was assessed with a single question from the Medical Outcomes Survey–Short Form– 12 Version 2 (SF-12v2): “In general, would you say your health is: excellent, very good, good, fair, or poor” [15].

For the ADHD group, the effect of ADHD on functioning was evaluated using a series of 6 questions, rated on a 5-point scale ranging from strongly disagree (1) to strongly agree (5). The 6 questions were as follows. ADHD has negatively impacted my: (1) ability to achieve success in my career/work life, (2) relationship with my spouse or significant other, (3) relationships with other people in my life, (4) self-esteem, (5) overall physical health, and (6) overall mental health.

The impact of health problems (any physical or emotional problem or symptom) on work productivity and general activity was measured using the Work Productivity and Activity Impairment Questionnaire (WPAI) [16]. The WPAI assesses the domains of absenteeism (time missed from work), presenteeism (impairment of work productivity while at work), overall work impairment (a combination of absenteeism and presenteeism), and activity impairment (impairment in ability to engage in regular activities) over the previous 7 days. Results for each domain are expressed as a percentage, with higher scores indicating a greater degree of impairment.

Health care resource use was assessed based on self-report. Participants were asked how many times they had visited various types of healthcare providers in the past 6 months. In addition, participants were asked how many times they had been to the emergency room or hospital for their medical condition in the past 6 months.

Comparisons between the ADHD and non-ADHD control groups were made using chi-square tests for categorical variables and t-tests for continuous variables. All analyses were completed using SAS version 9.2 (SAS, Inc., Cary, NC).

## Results

### Participants

A total 184 individuals from Japan were included in the analysis; 84 individuals from the online survey met inclusion criteria for the ADHD group and 100 individuals were included in the non-ADHD control cohort. Demographic characteristics were similar between the two groups, with the exception of age and gender. Participants in the ADHD group were on average slightly younger and had higher proportion of males (Table 1).

**Table 1:** Demographic Characteristics.

	Diagnosed ADHD^a^	Control
Variable	N = 84	N = 100
Age, mean (SD)	37.1 (9.2)	41.8 (12.2)^b^
Male, n (%)	57 (67.9%)	49 (49.0%)^b^
Married, n (%)	39 (46.4%)	50 (50.0%)
Education, n (%)		
<High School	6 (7.2%)	1 (1.0%)
High School Diploma	28 (33.3%)	34 (34.0%)
2 Year College	7 (8.3%)	18 (18.0%)
4 year College	34 (40.5%)	38 (38.0%)
Graduate Degree	9 (10.7%)	9 (9.0%)
Income, n (%)		
≤ 2,999,999 yen	22 (26.2%)	13 (13.0%)
3,000,000 to 6,999,999 yen	30 (35.7%)	33 (33.0%)
≥ 7,000,000 yen	27 (32.1%)	39 (39.0%)
No answer	5 (6.0%)	15 (15.0%)^b^
Employed, n (%)	66 (78.6%)	68 (68.0%)

^a^Patients reported being diagnosed with ADHD by a physician

^b^p < 0.05

### Health Status and Burden

**Fig 1:**
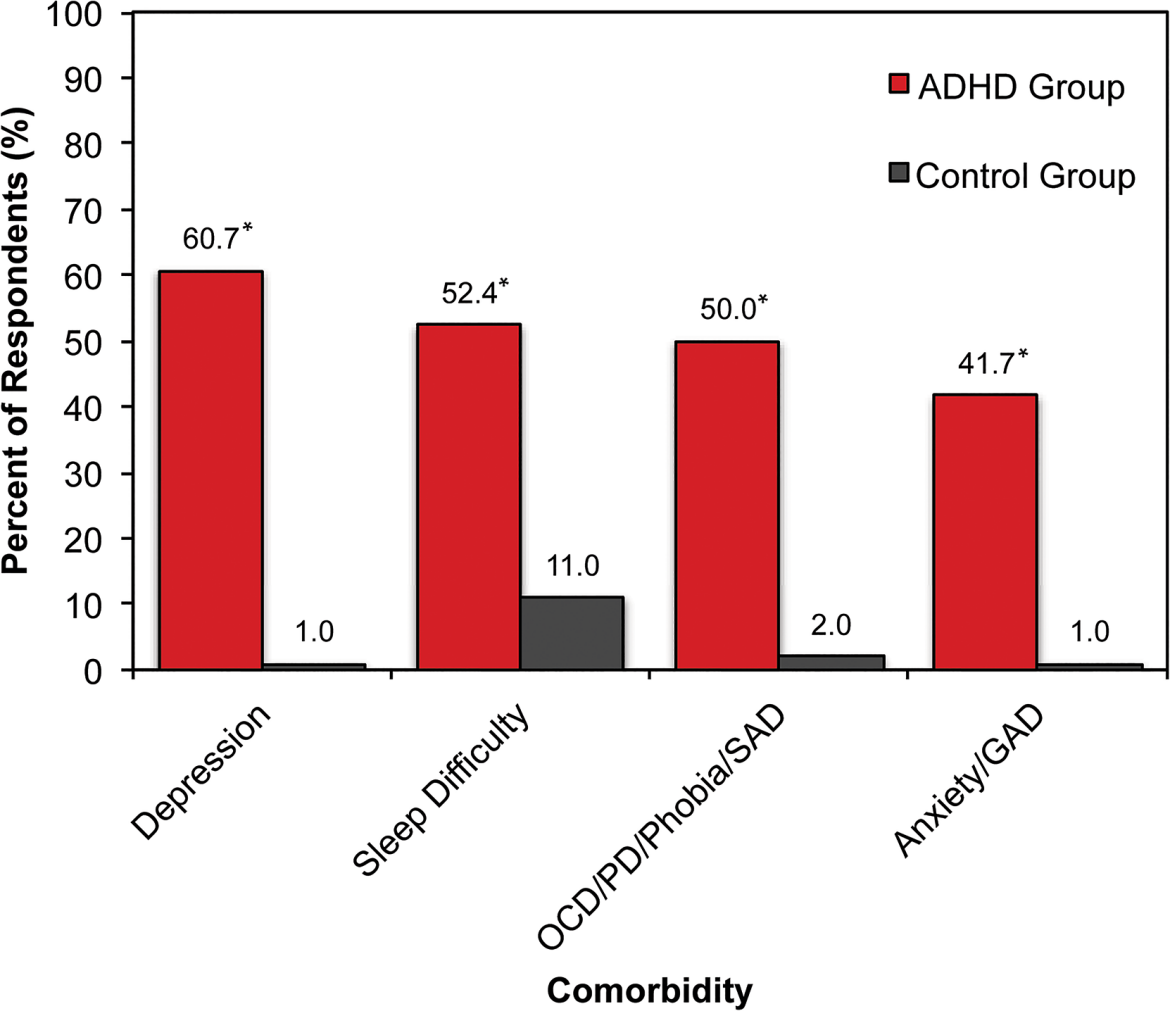
Comorbid Central Nervous System-related Conditions Reported over the Past 12 Months. In this self-report survey, the ADHD group consisted of patients who reported being diagnosed with ADHD by a physician. The ADHD group reported a significantly greater number of comorbid CNS-related conditions when compared to the non-ADHD control group. In particular, depression and general anxiety in the ADHD group occurred at a rate that was approximately 60- and 40-fold greater than non-ADHD controls, respectively. Abbreviations: ADHD = attention-deficit/hyperactivity disorder; CNS = central nervous system; GAD = Generalized Anxiety Disorder; OCD = Obsessive-Compulsive Disorder; PD = Panic Disorder; SAD = Social Anxiety Disorder. *p < 0.05.

Over the past 12 months, comorbid conditions reported significantly more frequently in the ADHD group than in the control group included depression, sleep difficulty, and anxiety-related disorders (such as Generalized Anxiety Disorder, Obsessive-Compulsive Disorder, Panic Disorder, phobias, and Social Anxiety Disorder). The most common comorbid condition reported by the ADHD group was depression (Fig 1). Specific to the ADHD group, 14.3% reported being diagnosed with alcoholism and 9.5% with substance use/abuse (other than alcohol), prior to being diagnosed with ADHD.

**Fig 2:**
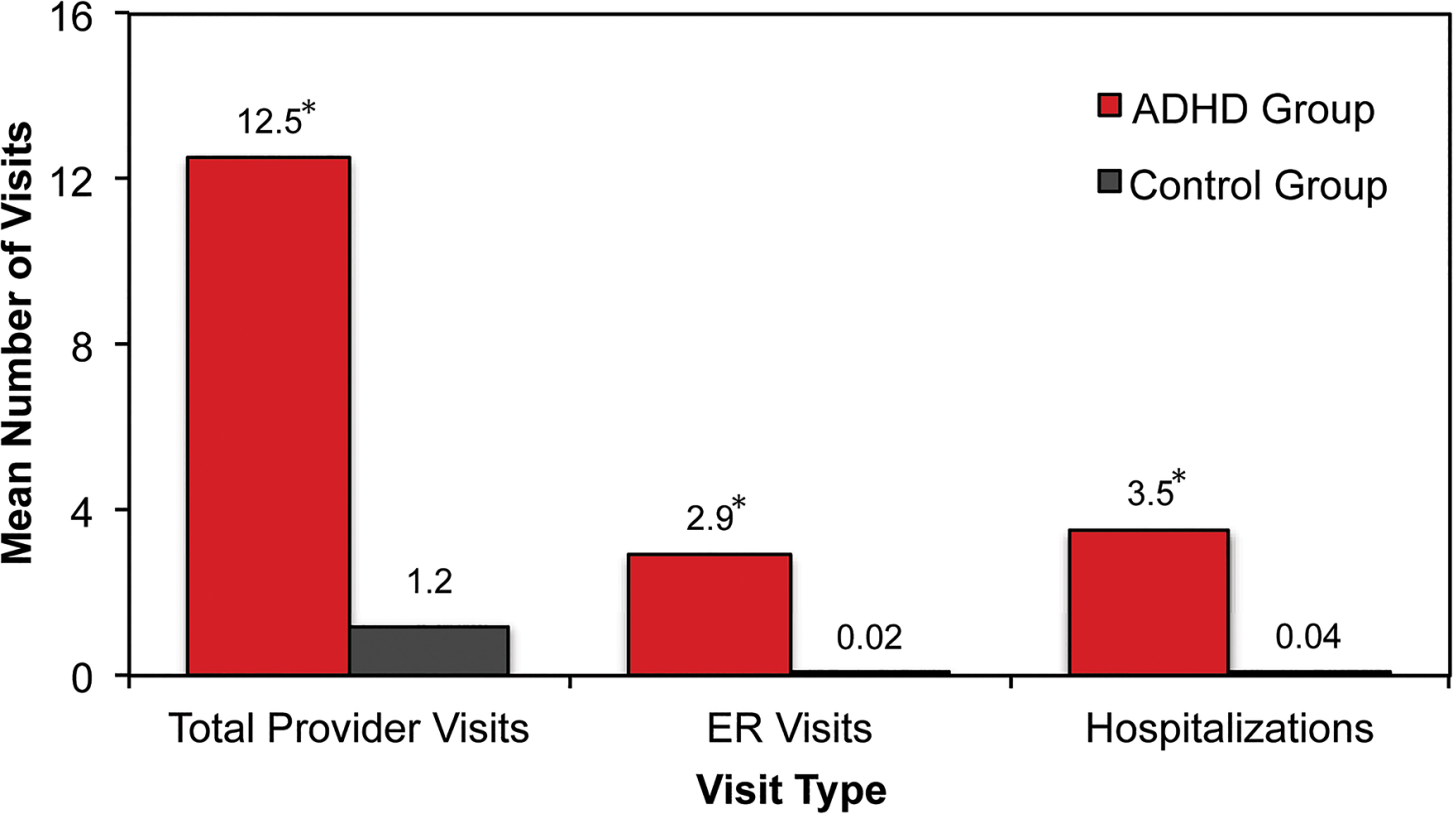
Healthcare Visits over the Past 6 Months. In this self-report survey, the ADHD group consisted of patients who reported being diagnosed with ADHD by a physician. Participants in the ADHD group had significantly more healthcare provider visits, emergency room visits, and hospitalizations when compared to the non-ADHD control group. The ADHD group reported total physician visits at a rate that was 10 times greater, and emergency room visits and hospitalizations at a rate that was approximately 3 times greater than the control group. Abbreviation: ADHD = attention-deficit/hyperactivity disorder; ER = emergency room. *p < 0.05.

Participants in the ADHD group also utilized significantly more healthcare resources over the previous 6 months, as measured by total healthcare provider visits, emergency room visits, and hospitalizations (Fig 2).

**Fig 3:**
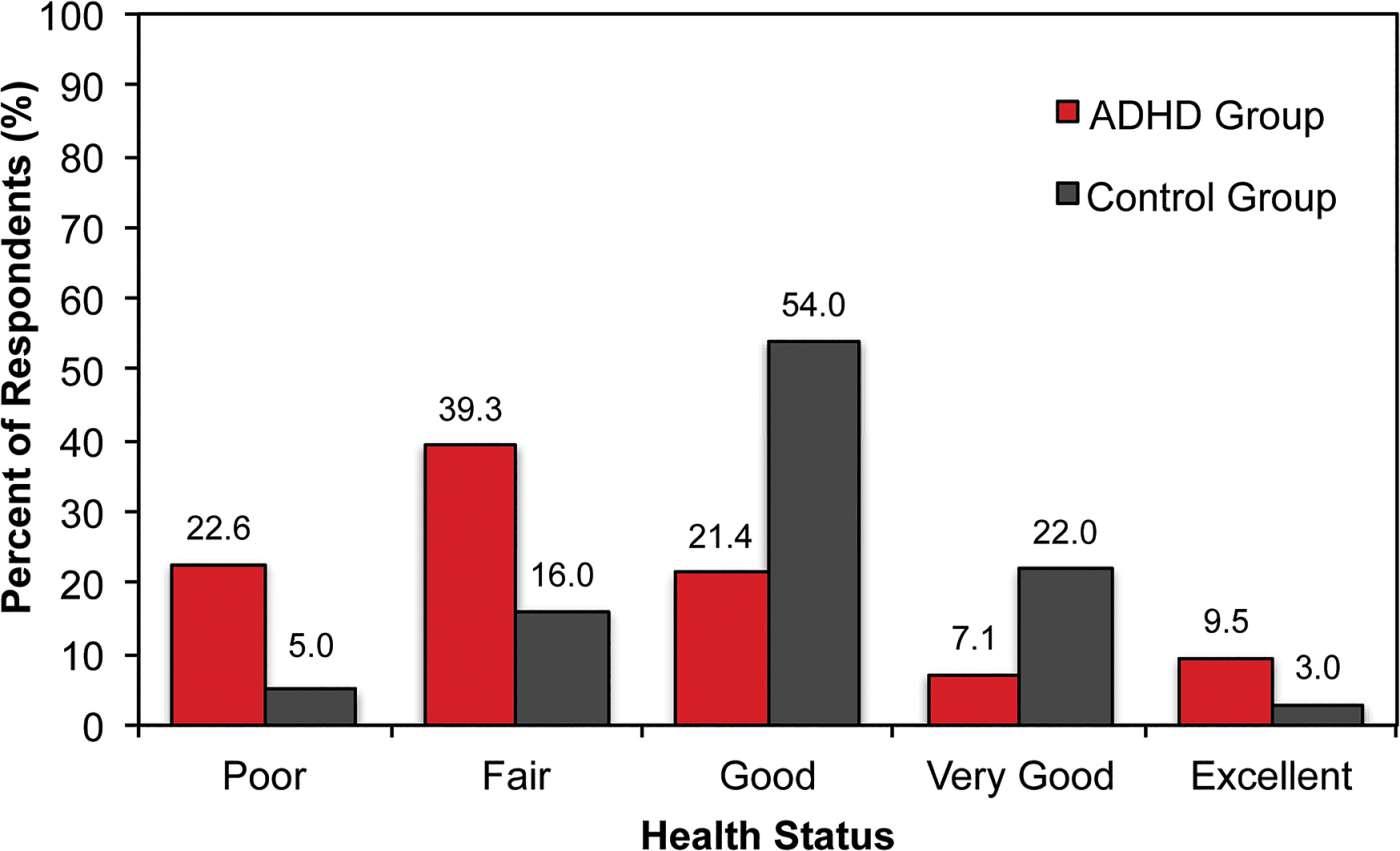
Participants Self-rated General Health Status. In this self-report survey, the ADHD group consisted of patients who reported being diagnosed with ADHD by a physician. The mean self-rated health status was 3.6 (SD 1.2) for the ADHD group and 3.0 (SD 0.8) for the non-ADHD control group (p < 0.05). Nearly 3 times as many participants in the ADHD group perceived their health status as being either “poor” or “fair” compared to the control group. Abbreviation: ADHD = attention-deficit/hyperactivity disorder.

Consistent with the greater frequency of visits to a healthcare provider, the ADHD group also rated their health status lower than the control group. The mean self-rated health status for the ADHD group and control group was 3.6 (standard deviation [SD] 1.2) and 3.0 (SD 0.8), respectively (p < 0.05), with a higher score indicating worse overall health. The modal rating for the ADHD group was “Fair” while the modal rating for the control group was “Good” (Fig 3). In the ADHD group, 62% rated their health as either poor or fair, compared to 31% in the control group.

### Occupational and Social Impact

With regard to occupational functioning, as assessed by the WPAI, participants in the ADHD group reported a higher rate of absenteeism over the past 7 days when compared to the control group (17.8% vs 2.0%; p < 0.05). In addition, health problems affected work productivity in the ADHD group to a greater extent than the control group as measured by the presenteeism scale (58.2% vs 18.2%; p < 0.05). Taking into account both absenteeism and presenteeism, the mean work impairment score was significantly greater for the ADHD group (64.7; SD 25.1) than for the control group (18.6; SD 22.4; p < 0.05).

According to the responses on the WPAI, the impact of health problems was not limited to work productivity. In the same time period, participants in the ADHD group also reported that their health problems affected their ability to engage in regular daily activities to a greater degree than reported by the control group. The activity impairment score was 59.2% for the ADHD group and 18.3% for the control group (p < 0.05).

Specific to the ADHD group, respondents reported that their ADHD symptoms negatively impacted their relationships and self-esteem (Table 2). The majority of participants in the ADHD group either agreed or strongly agreed that ADHD had negatively impacted their relationships at work (n = 64; 76.2%), relationships with their significant other (n = 58; 69.0%), other relationships (n = 65; 77.4%), and self-esteem (n = 62; 73.8%).

**Table 2:** Rating of ADHD Impact on Relationships and Self-esteem.

	Participant Response n (%)				
Negative Impact Domain	Strongly Disagree	Disagree	Neither	Agree	Strongly Agree
Relationships at work	2 (2.4%)	2 (2.4%)	13 (15.5%)	28 (33.3%)	36 (42.9%)
Relationship with significant other	2 (2.4%)	1 (1.2%)	18 (21.4%)	33 (39.3%)	25 (29.8%)
Other relationships	2 (2.4%)	5 (6.0%)	12 (14.3%)	32 (38.1%)	33 (39.3%)
Self-esteem	2 (2.4%)	5 (6.0%)	15 (17.9%)	26 (31.0%)	36 (42.9%)

## Discussion

The results from this study underscore the significant impact of adult ADHD on individuals in Japan with this condition in terms of overall health, as well as functioning in social and occupational settings. Japanese adults with ADHD reported a significantly greater number of comorbid central nervous system (CNS)-related conditions. In the presence of ADHD, depression and general anxiety occurred at particularly high rates that were approximately 60- and 40-fold greater than non-ADHD controls, respectively (Fig 1). The rate of depression reported in the non-ADHD group was very low (1%), leading to concerns that the differences between the two groups might be exaggerated. However, the prevalence for the non-ADHD group found in this study is consistent with another study conducted in Japan that reported a 12-month prevalence rate of 3% for depression [17].

Furthermore, individuals in the ADHD group utilized substantially more healthcare resources, as measured by visits to a physician, emergency room visits, and hospitalizations. In fact, the ADHD group reported visits to physicians at a rate that was 10 times higher than the control group, and approximately 3 times more visits to the emergency room and hospitalizations (Fig 2). Given these results, it is not surprising that ADHD was associated with a much lower perception of overall health, with nearly 3 times as many participants in the ADHD group perceiving their health status as being either “poor” or “fair” compared to the control group (Fig 3).

Significant impairments in occupational and social functioning were also attributed to the symptoms of ADHD. Individuals with ADHD missed more work, and reported being less productive while at work, than individuals in the control group. In fact, the mean overall work impairment score for the ADHD group was over 3 times higher compared to the control group. Furthermore, nearly 75% of participants in the ADHD group either “agreed” or “strongly agreed” that ADHD had a negative impact across relationships with their spouse, coworkers, and others.

The negative impact associated with adult ADHD found in this sub-analysis of Japanese patients is consistent with the overall results from the larger study that included Europe, Australia, and the United States [14]. Adults with ADHD across these geographies exhibited higher rates of CNS-related disorders, health-related productivity impairment at work, and healthcare utilization when compared to non-ADHD controls. The pattern of increased comorbidities, losses in work productivity, and higher rates of healthcare resource utilization found in this study are also consistent with the published literature. The associations between ADHD and CNS-related disorders observed here, particularly depression and anxiety, were observed in an Australian study that reported a significantly higher proportion of depression and anxiety symptoms in this population [6]. With the higher rates of anxiety and depression, presumably this would correspond with an increased use of medications to treat these conditions, and therefore result in an additional burden on healthcare costs. Combined with the higher rates of overall healthcare utilization for the ADHD group found here, these results suggest that the incremental costs related to ADHD in Japan may be significant, as was reported in a literature review for the United States [12].

In regard to comorbidities of CNS-related disorders, higher rates of Substance Use Disorders (SUD) in ADHD patients (12.5%) have been reported previously in the literature [7]. The rates of alcoholism and other substance abuse reported in this study (14.3% and 9.5%, respectively) were consistent with this prior estimate. The degree to which proper treatment of ADHD may impact the expression of these related disorders warrants further investigation.

In the present study, the disruption associated with ADHD in occupational and social settings reflects the findings from other parts of the world as well. A consistent pattern starts to emerge regarding lost productivity and difficulties in relationships with family, friends, and co-workers. Küpper and his colleagues reviewed the literature (limited to articles published in English or German) to assess the negative effects of ADHD in adults on work productivity and occupational health [10]. The authors conclude that absenteeism and lost productivity in adults with ADHD has a substantial economic impact. Furthermore, the narratives elicited from 14 focus groups across North America and Europe, uncovered difficulties in a wide array of relationships that included parents, partners, friends, work colleagues, and acquaintances [4]. The participants in this study also commonly reported seeing themselves as being different from others and feeling “not normal” or “wrong” as a result [4]. This negative perception of self for these individuals matches the findings reported here, in that nearly three quarters of the participants with ADHD indicated that ADHD had negatively impacted their self-esteem.

The results from this study add to, and are consistent with, the one known study regarding the consequences of ADHD adult in Japan in which ADHD was associated with increased stress and more frequent visits to a healthcare provider [8].

ADHD in adults is consistently associated with negative outcomes regarding relationships, work productivity, and healthcare utilization. Therefore, it is not surprising that they also tend to suffer from depression and anxiety. Proper diagnosis, and identifying the appropriate treatment and support are essential to help adults with ADHD improve their lives and also offset the costs associated with lost productivity and overall healthcare utilization.

### Limitations

Information from this study was obtained through self-report by online survey respondents, rather than from clinical or experimental data. Therefore, it was not possible to verify demographic and behavioral information provided by participants, nor corroborate the diagnosis of ADHD (or the absence thereof). In addition, the ADHD and control groups were not matched on age and gender.

## Conclusions

When compared to adults not suffering from ADHD, adults from Japan with ADHD face a substantial burden of illness including lower overall health status, increased number of comorbidities, greater healthcare utilization, and significant health-related occupational and social impairment. The results from this study add to the limited information available regarding the impact of adult ADHD specific to Japan. Additional research is needed to develop a better understanding of both the consequences and treatment approaches for Japanese adults with ADHD.
